# Four weeks of probiotic supplementation reduces GI symptoms during a marathon race

**DOI:** 10.1007/s00421-019-04136-3

**Published:** 2019-04-13

**Authors:** Jamie N. Pugh, Andy S. Sparks, Dominic A. Doran, Simon C. Fleming, Carl Langan-Evans, Ben Kirk, Robert Fearn, James P. Morton, Graeme L. Close

**Affiliations:** 1 Research Institute for Sport and Exercise Sciences, Liverpool John Moores University, Tom Reilly Building, Byrom St Campus, Liverpool, L3 3AF UK; 2 Sport Nutrition and Performance Research Group, Department of Sport and Physical Activity, Edge Hill University, Ormskirk, UK; 3 Royal Cornwall Hospital, Truro, UK; 4 School of Health Sciences, Liverpool Hope University, Liverpool, UK; 5 Homerton University Hospital NHS Foundation Trust, London, UK

**Keywords:** Marathon, Probiotics, Gastrointestinal, Exercise performance

## Abstract

**Purpose:**

To evaluate the effects of probiotic supplementation on gastrointestinal (GI) symptoms, circulatory markers of GI permeability, damage, and markers of immune response during a marathon race.

**Methods:**

Twenty-four recreational runners were randomly assigned to either supplement with a probiotic (PRO) capsule [25 billion CFU *Lactobacillus acidophilus* (CUL60 and CUL21), *Bifidobacterium bifidum* (CUL20), and *Bifidobacterium animalis* subs p. *Lactis* (CUL34)] or placebo (PLC) for 28 days prior to a marathon race. GI symptoms were recorded during the supplement period and during the race. Serum lactulose:rhamnose ratio, and plasma intestinal-fatty acid binding protein, sCD14, and cytokines were measured pre- and post-races.

**Results:**

Prevalence of moderate GI symptoms reported were lower during the third and fourth weeks of the supplement period compared to the first and second weeks in PRO (*p* < 0.05) but not PLC (*p* > 0.05). During the marathon, GI symptom severity during the final third was significantly lower in PRO compared to PLC (*p* = 0.010). The lower symptom severity was associated with a significant difference in reduction of average speed from the first to the last third of the race between PLC (− 14.2 ± 5.8%) and PRO (− 7.9 ± 7.5%) (*p* = 0.04), although there was no difference in finish times between groups (*p* > 0.05). Circulatory measures increased to a similar extent between PRO and PLC (*p* > 0.05).

**Conclusion:**

Probiotics supplementation was associated with a lower incidence and severity of GI symptoms in marathon runners, although the exact mechanisms are yet to be elucidated. Reducing GI symptoms during marathon running may help maintain running pace during the latter stages of racing.

## Introduction

Gastrointestinal (GI) symptoms are widely reported in athletes participating in prolonged endurance events (Costa et al. 2017a). In marathon running, 27% of recreational runners report moderate or more severe GI symptoms during a race (Pugh et al. 2018). The pathogenesis of such symptomology is still poorly understood, although it is likely multifactorial in nature. The exercise-induced reduction in splanchnic blood flow is well characterised (Otte et al. 2001) and results in dysregulation of the intestinal barrier. This likely leads to endotoxemia and an immunological response, which has been associated with GI symptoms during ultra-endurance events (Jeukendrup et al. 2000). Carbohydrate (CHO) intake during exercise is also suggested to be a potential causative and or aggravating factor, due to malabsorption when consumed in excess (de Oliveira and Burini 2014), although tolerance levels may differ for individuals (Costa et al. 2017b). There may also be a predisposition to symptoms, as individuals with a history of recurrent exercise-associated GI symptoms, appear to suffer greater prevalence and severity of symptoms during exercise (Ter Steege et al. 2008). However, due to the range of symptom types (each with their own unique aetiology) coupled with differences in study methodologies to assess GI symptoms, studies to date have yet to find a single mechanism. Nonetheless, GI symptoms during marathon running remain detrimental to exercise performance in recreational and elite runners (Pugh et al. 2018) hence potential strategies to reduce such remains an attractive area of research.

One potential strategy to reduce GI symptoms during endurance exercise is probiotic supplementation (Roberts et al. 2016). While probiotics can relieve lower GI symptoms in irritable bowel syndrome IBS (Hungin et al. 2018), there is less consensus with regard to their efficacy in modulating exercise-associated GI symptoms. A reduction in the duration of GI symptoms was noted in a group of recreational runners 2 week post-marathon race following a single strain probiotic supplementation, although only severe symptoms (diarrhea, vomiting, and stomach ache) were recorded and no differences were found during the period of supplementation (Kekkonen et al. 2007). The severity of GI symptoms during training in novice triathletes was also reduced when supplementing with a multi-strain probiotic, with subsequent reductions in GI permeability following an Ironman distance triathlon race compared to placebo (Roberts et al. 2016). However, permeability was assessed 6 day post-race and in-race GI symptoms were not assessed. Potential mechanisms by which symptoms could be ameliorated include modulation of CHO absorption and oxidation (Rooj et al. 2010), and attenuation of exercise-induced GI damage or permeability (Anderson et al. 2010; Lamprecht et al. 2012).

There are many circulatory markers of GI dysfunction that have been assessed post-exercise. Indirect measures of GI permeability utilise dual sugar probes, such as lactulose and rhamnose (Pugh et al. 2017b), while intestinal-fatty acid binding protein (I-FABP) has often been used as a sensitive marker to intestinal mucosal damage (Pugh et al. 2017a; Van Wijck et al. 2011). However, the relationship between such markers and exercise-induced GI symptoms is unclear. Downstream circulatory markers of immune activation and inflammatory response have been assessed following competitive endurance exercise (Moore et al. 1995; Nieman et al. 2006), although to date, only one marker has been associated with the occurrence of GI symptoms. Plasma soluble cluster of differentiation 14 (sCD14) is a co-receptor of lipopolysaccharide (LPS) and a marker of monocyte activation (Shive et al. 2015). Post-race plasma concentrations of sCD14 were increased to a greater extent in ultramarathon runners reporting symptoms of nausea relative to those without (Stuempfle et al. 2016). Therefore, sCD14 warrants further investigation following endurance exercise.

Given the prevalence of GI issues in endurance athletes and the associated effects on exercise performance and perceived enjoyment, the purpose of this study was to evaluate the effects of probiotic supplementation on GI symptoms during marathon training and racing. We hypothesised that GI symptoms during training and a marathon race would be less frequent and less severe with probiotic supplementation. In addition, it was hypothesised that probiotic supplementation would attenuate circulatory markers of exercise-induced GI permeability, damage, and immunological response.

## Methods

A total of 24 runners (20 male, 4 female) participated in the study. All participants were required to have run a marathon race quicker than 5 h within the previous 2 years. Participant characteristics are presented in Table 1. All participants were free of medications, such as NSAIDs, antidepressants, or diuretics, nutritional supplements and any history of GI-related medical issues (IBS or abdominal surgery). After explaining the nature and risks of the experimental procedures to the participants, their informed written consent was obtained. The study was approved by the Ethics Committee of Liverpool John Moores University.

**Table 1:** Participant characteristics

	PLC		PRO	
	Mean ± SD	Range	Mean ± SD	Range
Age (years)	36.1 ± 7.5	29–50	34.8 ± 6.9	22–43
Height (cm)	175.4 ± 11.1	152–186	179.0 ± 6.3	168–190
Body mass (kg)	73.5 ± 11.3	48–95	76.5 ± 9.4	61–92
$\dot{V}$O_2peak_ (mL kg min^−1^)	56.4 ± 8.6	47.2–70.0	57.6 ± 8.0	48.1–66.7
LT (km h^−1^)	11.9 ± 1.9	9–16.0	12.3 ± 1.9	10–15.5
Most recent marathon time (min)	220 ± 40	150–283	222 ± 46	152–315

Values are mean ± SD. Differences between groups for all measures were not significant

### Baseline testing

At least 4 weeks before the marathon, and prior to the supplement period, participants visited the laboratory and completed the Gastrointestinal Symptom Rating Scale (Svedlund et al. 1988) to assess baseline GI symptoms. Participants then completed an incremental running test to determine lactate threshold (LT) and peak oxygen uptake ($\dot{V}$O_2peak_) as previously described (Jones 2006). Briefly, participants ran a minimum of six stages on a motorized treadmill (HP Cosmos Saturn, Traunstein, Germany). Each stage was 3 min in duration and interspersed with 30 s breaks to allow blood sampling. Running speed was increased by 1 km h^−1^ at the end of each stage, until runners reached volitional exhaustion.

### Supplement period

After baseline testing, in a double-blind, randomised and matched-pairs design, participants underwent a 28 day period of supplementation consuming either a commercially available probiotic (PRO) or a visually identical placebo (PLC). Participants also consumed an additional supplement capsule on the morning of the race, 2 h before the start. Participants were matched according to their most recent marathon performance (PRO: 222 ± 46 min; PLC 220 ± 40 min) and body mass (Table 1). The probiotic supplement capsules contained the active strains *Lactobacillus acidophilus* CUL60, *L. acidophilus* CUL21*, Bifidobacterium bifidum* CUL20, and *Bifidobacterium animalis* subsp*. Lactis* CUL34 (Proven Probiotics Ltd, Port Talbot, Wales). The minimum concentration was 25 billion colony-forming units (CFU) per capsule. The placebo capsules were visually identical and consisted of cornstarch only (Proven Probiotics Ltd, Port Talbot, Wales). Participants were instructed to swallow the capsule daily after their first meal. The randomization code was held by a third party, unlocked for analyses upon sample analysis completion. During the supplementation period, participants were instructed to refrain from all probiotic foods (i.e., fermented yogurts). For the full 28 day supplement period, participants were required to complete a daily training and GI symptom diary. Six GI symptoms were included: bloating, nausea, urge to vomit (upper GI), flatulence, urge to defecate, and stomach cramps (lower GI). Symptoms were scored from 0 to 10 on a visual analogue scale. Symptoms scored ≥ 4 were classified as ‘moderate’ or worse and these data were summed during the supplementary period.

### Marathon race

During the 24 h before the race, participants consumed a standardized high CHO, low fibre diet [per kg body mass: 8.0 g CHO (0.28 g fibre); 2.0 g protein; 1.0 g fat]. Compliance to the diet was confirmed with food diaries and the remote food photography method (Martin et al. 2009). After an overnight fast, participants reported to the laboratory at ~ 07:00 h and resting blood samples were taken. Participants were then provided a standardized breakfast [572 kcal; 128 g CHO (4.4 g fibre), 7 g protein, 3.5 g fat, and a minimum of 500 mL water] before a pre-race venous blood sample was collected. Participants performed self-selected warm-ups before a race briefing to reiterate in-race nutrition and subjective measures. The race started at 12:00 pm. Runners ran the 42,195 m race on a synthetic 400 m outdoor track (105.48 laps) which was in close proximity to the laboratory. Weather conditions throughout the race were: temperature: 16–17 °C; wind speed: 8–16 km h^−1^; precipitation: 0 mm. During the race, heart rate was monitored throughout (Firstbeat Sports©, Jyväskylä, Finland) and subjective ratings of perceived exertion (RPE) (Borg 1970) were recorded every 15 min. Each 400 m lap time was recorded using electronic chips and a timing mat (BibTag System, MYLAPS, USA). Global GI discomfort was assessed every 15 min using a modified Likert scale (Nieman et al. 2006). These physiological and symptomology data were consolidated and reported as the mean of each third of the race distance completed, given that reductions in average running pace and reporting of ‘hitting the wall’ are seen after 25–30 km (Santos-Lozano et al. 2014; Buman et al. 2009), and glycogen depletion is theorised to occur between 32 and 40 km (Locksley 1980).

### In-race nutrition

Participants consumed one 60 mL CHO gel (SIS Isotonic Gel, Blackburn, UK) and 200 mL of water 10–15 min before the start of the race and one 60 mL CHO gel with 200 mL of water 40 min after the start of the race and subsequently every 20 min for the remainder of the race. Gels consisted of 22 g maltodextrin and 0.01 g sodium. This provided an average of 66 g h^−1^ CHO in 180 mL and 600 mL h^−1^ of water during the race, a strategy that has been shown to improve performance in non-elite runners relative to a self-selected strategy (Hansen et al. 2014). To familiarize with this nutritional strategy, participants were informed of the strategy and provided with identical gels to practice this during their two longest training runs during the prior 4 week supplementation period. This was diarized in the GI symptom and training diary.

### Post-race

Blood samples were collected immediately post-race for later analysis. Participants were then asked to complete a more detailed questionnaire (adapted from Pfeiffer et al. 2012) to assess any specific symptoms of GI discomfort, including: bloating, flatulence, stitch, belching, nausea, urge to vomit, urge to defecate, and stomach cramps. These were scored on a 10-point scale (0 = no pain and 10 = worst possible pain) with a score > 4 being regarded as moderate. To ensure understanding, specific symptoms were explained and described to participants. This same scale was used in the daily GI symptom diary used during the supplementation period.

### Blood analysis

Blood samples were collected into vacutainers containing EDTA, lithium heparin, and serum separation tubes. From whole blood samples, duplicate measures of haematocrit (Hawksley micro-haematocrit reader, Sussex, UK) and haemoglobin (Haemocue, Sussex, UK) were taken. Serum samples were allowed to clot for 1 h at room temperature, while EDTA and lithium heparin samples were immediately stored on ice, following which all samples were centrifuged for 15 min at 1500 RCF at 4 °C. Serum and plasma were manually extracted and stored at − 80 °C until required for analysis. Samples were analysed for plasma glucose, intestinal-fatty acid binding protein (I-FABP), sCD14, interleukin-6 (IL-6), IL-8, IL-10, and serum cortisol. Post-race sample concentrations were corrected for plasma volume changes as described by Dill and Costill (1974).

**Fig. 1:**
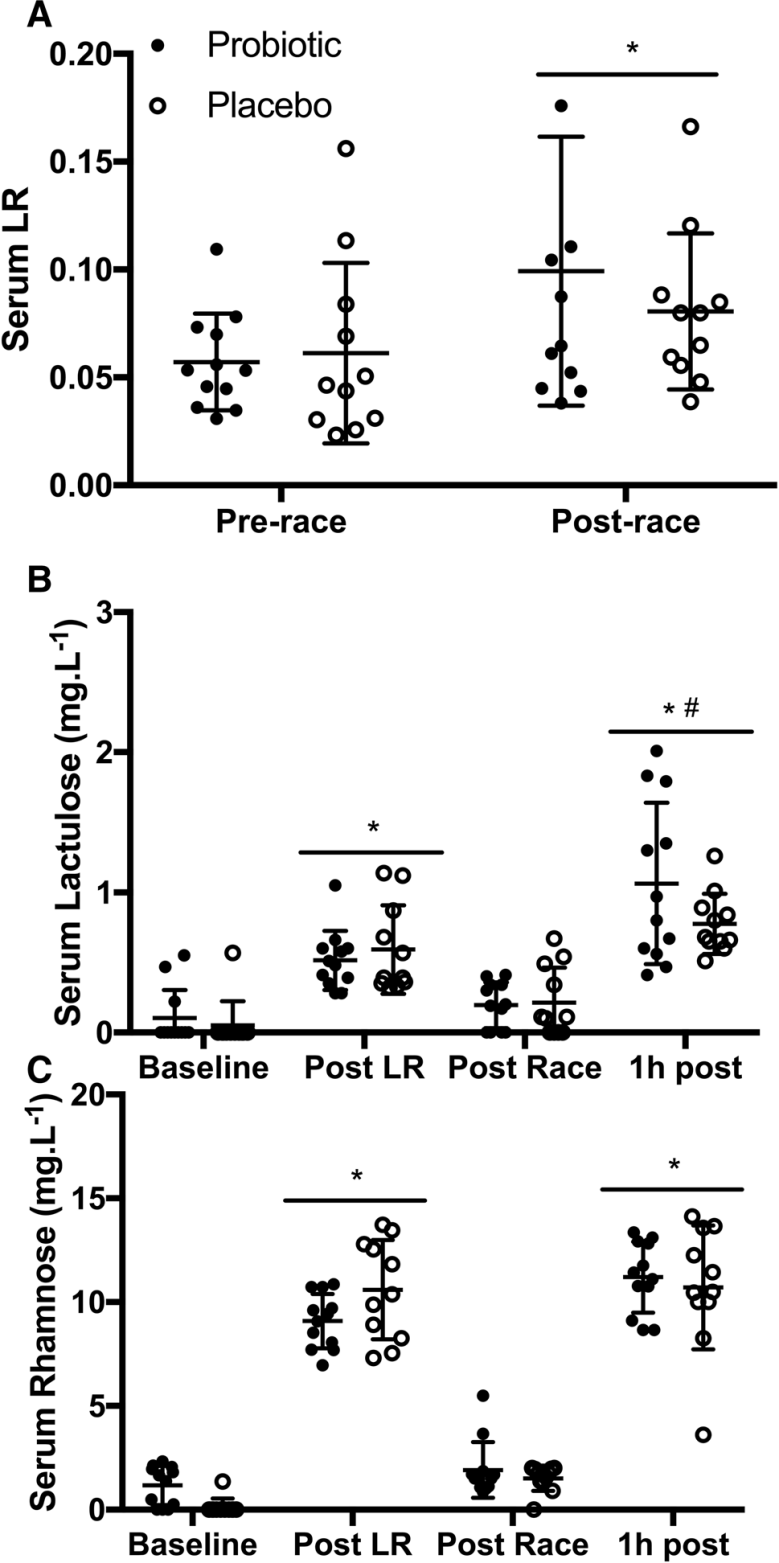
Serum lactulose:rhamnose ratio (**a**), laculose (**b**), and rhamnose concentrations (**c**) at each sampling point. *Significant difference from pre-race (*p* < 0.05), ^#^significant difference to post LR (*p* < 0.05)

Intestinal permeability was assessed by analysing serum samples using a previously published protocol (Fleming et al. 1996), with the modification of using rhamnose instead of mannitol as the monosaccharide probe. Briefly, at baseline, a 50 mL sugar probe solution (5 g lactulose, 2 g rhamnose) was consumed and the ratio of the sugars was measured from serum samples 60 min after ingestion. A second identical probe was consumed immediately post-race and serum samples taken after 60 min for LR assessment. Pilot testing within our laboratory showed that after consuming the LR probe, serum lactulose and rhamnose concentrations had returned to baseline 7 h post ingestion and that a second LR probe at this time was able to detect post-exercise LR increase relative to a morning resting sample, which was also demonstrated on race day (Fig. 1b, c).

Concentrations of I-FABP from EDTA plasma were determined using an ELISA (Hycult Biotechnology, Uden, The Netherlands; detection window 47–5000 pg mL^−1^) according to the manufacturer’s instructions. The coefficient of variation (CV) was 8.0% for between-sample duplicates. Plasma sCD14 was measured with a commercial enzyme-linked immunosorbent assay kit (R&D Systems, Inc., Minneapolis, Minnesota, USA) according to the manufacturer’s instructions, with a CV of 5.9%. Cytokine concentrations were measured using cytometric bead array (CBA, BD Biosciences, San Diego, USA) for the cytokines IL-6, IL-8, and IL-10 using the manufacturer’s instructions with three bead populations with distinct fluorescence intensities coated with capture antibodies specific for IL-6, IL-8, and IL-10 proteins with analysis on a BD Accuri flow cytometer (Becton–Dickinson, Franklin Lakes, NJ, USA). Following acquisition of sample data using the flow cytometer, the sample results were generated in graphical and tabular format using the BD CBA Analysis Software. The combined coefficients of variation were 9.7%. Serum cortisol was measured using an ELISA kit according to the manufacturer’s instructions (Elecsys Cortisol assay, Cobas-Roche, UK), with a CV of 2.9%.

### Statistical analysis

Descriptive statistics were produced for all data sets to check for normal distribution indicated by Shapiro–Wilk test (accepted if *p* > 0.05). A two-factor mixed measures ANOVA was used to examine differences in LR, I-FABP, sCD14, cytokines, and cortisol with condition (PRO and PLC) and various timepoints as the independent variables. Where significant main effects and interactions were present, pairwise comparisons were performed using the Sidak test method. For physiological and symptomology measures, individual data points were consolidated and averaged for each third of the race distance covered. To evaluate data on GI symptoms, a nonparametric statistical approach was chosen, as scores on GI symptoms were mainly reported on the low end of the scale and not normally distributed. Symptom diary variables were compared with the use of Wilcoxon Signed Rank tests. GI symptom diary data were analysed as the sum of all moderate or worse symptoms (scale of 0–84; a maximum of 6 each day) and the total number of days in which any GI symptoms ≥ 4 were reported. Symptom scores during the race were compared with the use of Mann–Whitney nonparametric *U* test for independent data. Spearman rank-order correlation was used to analyse the relationship between GI symptoms, with post-exercise I-FABP, inflammatory cytokines, and sCD14 concentrations. All normally distributed data are presented as mean ± standard deviation (SD). Data not normally distributed are reported as median and range. *p* < 0.05 was considered statistically significant. Statistical analysis was conducted using the Statistical Package for the Social Sciences software programme (SPSS, version 23) and Prism statistical software (GraphPad Prism, version.7.0c, La Jolla, CA, USA).

## Results

### GI symptoms during supplementary period

Baseline assessment scores from the GSRS showed no differences in GI symptoms between groups for any individual symptom, lower GI, upper GI, or total GI symptom scores (data not shown). From GI symptom diaries, daily average scores for specific symptoms were low (< 2) in both PRO and PLC. However, there was a large range of scores for individual symptoms, with 17 participants reporting symptoms with moderate severity or worse during at least 1 day. Table 2 displays the median number of total symptoms scored as moderate (≥ 4) for upper and lower GI symptoms, as well as the number of days with one or more moderate symptom. Data are shown for the first 14 days and the second 14 days. For the probiotic group, there were significant reductions in the number of moderate symptoms reported as well as the number of days in which moderate symptoms were reported in the second two of supplementation compared to the first 2 weeks (Table 2). There were no differences in the placebo group.

**Table 2:** GI symptoms reported during days 1–14 and 15–28 days during supplementation

	PRO			PLC		
	Days 1–14	Days 15–28	Wilcoxon *p* value	Days 1–14	Days 15–28	Wilcoxon *p* value
Total number of GI scores ≥ 4	4 (0–25)	2 (0–16)	0.007*	5 (0–41)	11 (0–39)	0.336
Total number of upper GI scores ≥ 4	0 (0–10)	0 (0–10)	0.317	3 (0–8)	3 (0–6)	0.514
Total number of lower GI scores ≥ 4	3 (0–16)	1 (0–8)	0.007*	4 (0–11)	5 (0–12)	0.317
Number of days with one or more symptom scored ≥ 4	3 (0–12)	1 (0–6)	0.011*	5 (0–12)	5 (0–10)	0.579

Data are presented as median and range

*Significant difference between days 1–14 and 15–28 (*p* < 0.05) (Mann–Whitney *U* test)

### Race completion and sample collection

A total of 20 runners completed the marathon race. There was one drop-out from PRO and three from PLC. Participants were asked to self-describe their reason for drop-out. In PLC, participants withdrew from the race due to musculoskeletal injury (22.5 km of the race completed) and two due to severe GI discomfort (12.2 and 30.2 km). In the PRO group, withdrawal was due to reflux (13.4 km). Those runners who completed at least 50% of the total were included for analysis for all blood analysis, and all participants were included for GI symptom scores during training. Blood samples could not be obtained at any timepoint from one participant who completed the race. All athletes were 100% compliant to the in-race water prescription. Adherence to the gel consumption nutritional plan was high with only one gel missed by three participants (2 PLA and 1 PRO).

### Overview of marathon performance

Participants completed the marathon in times between 152 and 302 min (Table 3). For all physiological measures, there were no significant differences between PLC and PRO (*p* > 0.05). For HR and RPE, data are shown for each third of the race distance. For all participants, RPE increased from the first to the final third (*p* < 0.001) of the race. Post-race blood glucose concentrations were increased in both groups relative to pre-race samples (*p* < 0.001).

**Table 3:** Physiological responses to the marathon

Timepoint	PLC (*n* = 9)	PRO (*n* = 11)
Finish time (min)	247 ± 47	234 ± 38
Running speed (%LT)	91.3 ± 8.7	90.2 ± 9.1
Heart rate (bpm)		
1/3 of race	162 ± 9	156 ± 13
2/3	161 ± 15	160 ± 9
3/3	155 ± 19	161 ± 8
RPE		
1/3 of race	12.6 ± 0.7	12.5 ± 1.0
2/3	14.5 ± 0.9	14.8 ± 1.8
3/3	16.5 ± 1.7^a^	16.3 ± 2.5^a^
Blood glucose (mmol L^−1^)		
Pre	5.3 ± 0.6	5.2 ± 1.1
Post	7.5 ± 1.1^b^	8.4 ± 1.6^b^
Haemoglobin (g dL^−1^)		
Pre	14.2 ± 0.9	13.8 ± 1.6
Post	13.8 ± 1.9	13.9 ± 12
Haematocrit (%)		
Pre	42.5 ± 3.2	41.5 ± 3.7
Post	42.9 ± 4.2	42.6 ± 3.1
PV change (%)	2.1 ± 8.4	− 1.4 ± 6.8

Data are mean ± SD

^a^Significantly different to 1/3 race distance (*p* < 0.001)

^b^Significantly different to pre-race (*p* < 0.001)

**Fig. 2:**
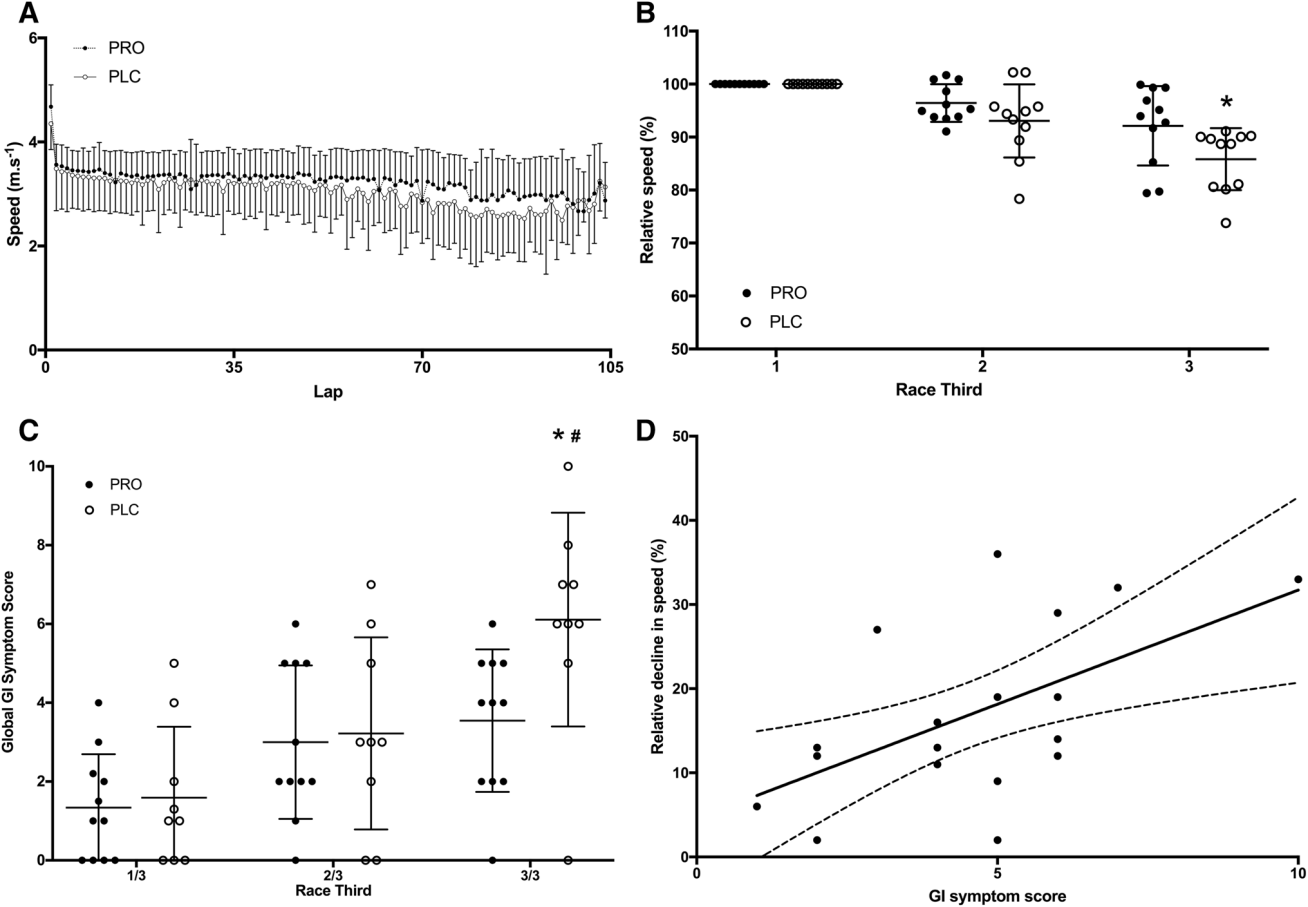
**a** Running speed during each lap of the race. **b** Average running speed during each third of the race, relative to first third. *Significant difference between PLC and PRO (*p* < 0.05). **c** Global GI symptom scores during each third of the race. *Significant increase compared to PRO. **d** Correlation between relative decline in speed and average global GI symptom scores during the final third of the race

Mean running speed during each lap for PLC and PRO is shown in Fig. 2a. Average running speeds were calculated for both groups during each third of the race and relative comparisons were made between each third (Fig. 2b). During the second third of the race, relative reductions in speed were 3.6 ± 3.5% for PRO and 6.9 ± 6.9% for PLC, although this was not significant (*p* = 0.165). During the final third, the reduction in average relative speed was greater in PLC (14.2 ± 5.8%) compared to PRO (7.9 ± 7.5%) (*p* = 0.03). This difference also remained significant between PLC (14.1 ± 6.1) and PRO (8.0 ± 6.7%) when only matched pairs were considered (*n* = 8 per group) (*p* = 0.04).

### Global GI symptoms during the race

During the race, global GI scores were averaged across each third (Fig. 2c). There were significant effects of both time (*p* < 0.001) and condition (*p* = 0.03). For time, there no significant differences between thirds for PRO. For PLC, there was a significant difference between GI symptom scores between the first (1.6 ± 1.8) and final third (6.1 ± 2.7) of the race (*p* < 0.001). While differences between conditions, groups were not significant for the first (*p* = 0.722) or second (*p* = 0.205) third of the race, GI symptoms were significantly lower in PRO compared with PLC during the final third of the race (*p* = 0.010) (Fig. 2c). When only matched pairs were considered (*n* = 8 per group), GI symptoms were still significantly higher in the final third (5.6 ± 2.9) of the race compared to the first (1.5 ± 2.0) for PLC (*p* = 0.001). The difference in GI symptoms scores between groups during the final third of the race only trended towards significance (*p* = 0.08) between PLC (5.6 ± 2.9) and PRO (3.25 ± 2.1). For both groups combined (*n* = 20), there was a significant correlation between average global GI score in the final third, and a reduction in average pace during this final third relative to the first third (*r* = 0.562, *p* = 0.010) (Fig. 2d).

### Circulatory markers of immune activation and GI dysregulation

**Fig. 3:**
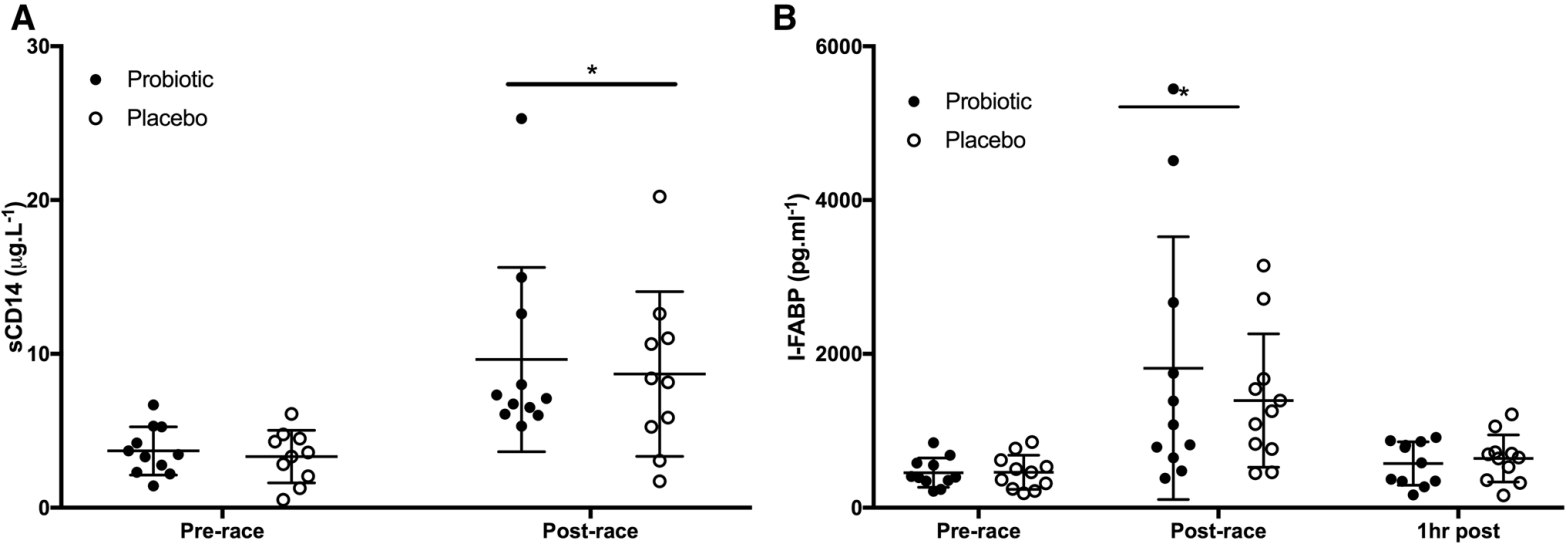
**a** Plasma sCD14 concentrations pre- and post-race for PRO (*n* = 11) and PLC (*n* = 10) groups. *Significant difference from pre-race (*p* < 0.05). **b** Plasma intestinal-fatty acid binding protein (I-FABP) pre-, post-, and 1 h post-race for PRO and PLC. *Significant difference from pre-race (*p* < 0.05)

Differences in sCD14 between PRO and PLC were not significant either pre- or post-race (Fig. 3a). For both groups, sCD14 was significantly increased post-race (PLC = 8.7 ± 5.1 µg mL^−1^, PRO = 9.6 ± 5.7 µg mL^−1^) compared to pre-race (PLC 3.3 ± 1.7 µg mL^−1^, *p* = 0.006; PRO 3.7 ± 1.6 µg mL^−1^, *p* = 0.022). Changes in pre-to-post-race sCD14 concentrations were significantly correlated with a global GI symptoms reported during the final third of the race (*r* = 0.546, *p* = 0.016).

Differences in LR (Fig. 1a) and I-FABP (Fig. 3b) were not significant at any timepoints between PRO and PLC. There was a significant effect of time for LR with a significant difference between pre- (PRO = 0.057 ± 0.022; PLC = 0.061 ± 0.042) and post-race values (PRO = 0.099 ± 0.062; PLC = 0.081 ± 0.036) (*p* = 0.040). Differences in LR pre-to-post-race were due to significant increases in serum lactulose concentration 1 h post-race compared to post LR (Fig. 1b), while there was no significant difference in serum rhamnose concentrations at these timepoints (Fig. 1c). For both groups, I-FABP was significantly increased post-race (PRO = 1814 ± 1708 pg mL^−1^; PLC = 1392 ± 867 pg mL^−1^) compared to pre-race (PRO 455 ± 190 pg mL^−1^; PLC 460 ± 221 pg mL^−1^) (*p* = 0.0004). The difference was not significant at 1 h post-race (*p* = 0.925). Post-race values for LR (*r* = − 0.250, *p* = 0.289), changes in pre-to-post-race LR (*r* = − 0.275, *p* = 0.24), and changes in pre-to-post-race concentrations of I-FABP (*r* = − 0.481, *p* = 0.075) did not significantly correlate with global GI symptom scores.

The concentrations of IL-6, IL-8, IL-10, and cortisol are presented in Table 4. There were no significant differences for either pre- or post-race concentrations between PLC and PRO (*p* > 0.05). For both groups, all measures increased from pre- to post-races (*p* < 0.001). Post-race values did not correlate with global GI symptom scores (*p* > 0.05).

**Table 4:** Pre- and post-exercise cytokine and cortisol concentrations for placebo and probiotic groups. Data are mean ± SD

	PLC		PRO	
	Pre-race	Post-race	Pre-race	Post-race
IL-6 (pg mL^−1^)	0.82 ± 0.74	13.58 ± 12.9^a^	1.01 ± 0.66	10.96 ± 7.86^a^
IL-8 (pg mL^−1^)	2.13 ± 2.03	12.11 ± 6.33^a^	2.24 ± 0.92	13.45 ± 11.18^a^
IL-10 (pg mL^−1^)	0.73 ± 0.64	5.78 ± 3.26^a^	0.91 ± 0.55	5.27 ± 3.91^a^
Cortisol (nmol L^−1^)	614 ± 119	1083 ± 196^a^	653 ± 155	1148 ± 242^a^

^a^Significant difference from pre-race (*p* < 0.001)

### GI symptoms assessed post-race

Mean scores for specific symptoms immediately post-race and 24 h post were all low for both PRO and PLC (≤ 2) with no significant differences between conditions for all symptoms. From these, total, upper, and lower GI symptom scores were calculated, with no significant differences between PRO and PLC (Table 5). All participants reported at least one symptom during the race and 24 h post-race (score ≥ 2), while 50% reported at least one moderate symptom (score ≥ 4). Correlations between measures of either any of the individual specific GI measures, or pooled totals (total score, lower GI score, and upper GI score) assessed post-race and global GI symptoms were all not significant (*p* > 0.05).

**Table 5:** GI symptoms measured immediately and 24 h post-race

	PLC	PRO
Immediately post-race		
Total GI symptom score	15 (3–51)	13 (0–37)
Upper GI symptom score	5 (0–30)	6 (0–16)
Lower GI symptom score	7 (0–21)	10 (0–31)
24 h post-race		
Total GI symptom score	12 (0–43)	16 (0–36)
Upper GI symptom score	4 (0–46)	6 (0–18)
Lower GI symptom score	5 (0–25)	7 (0–28)

Data are presented as median and range

## Discussion

The aim of the present study was to evaluate the effects of probiotic supplementation on gastrointestinal (GI) symptoms, circulatory markers of GI permeability and damage, cytokines and cortisol during a marathon race. Utilising an officially sanctioned track marathon race in a wide range of experienced marathon runners, we report that all runners experienced GI symptoms, with two athletes abandoning the race due to severe GI discomfort. Of note, there was an association between the subjective scores of in-race GI symptoms and reductions in running speed, especially towards the later stages of the race. We report for the first time that runners supplementing with probiotics reported fewer and less severe GI symptoms, both in training and during a marathon race when using standardized, recommended CHO loading and in-race CHO and hydration strategies. Those runners supplementing with probiotics also demonstrated less of a performance decrement (as evidenced by maintaining running speed) towards the end of the race. Taken together, our data suggest that supplementation with probiotics could have performance benefits during competitive endurance exercise through alleviation of GI symptoms.

In relation with the 4 week supplementation period, our data demonstrate that participants reported fewer moderate GI symptoms during training (as reported in a daily GI symptom diary), an effect that was observed after 2 weeks of probiotic supplementation. In subsequently assessing global GI symptoms using a visual analogue scale during the marathon race itself, we were also able to report, in real time, the development of GI symptoms throughout the race. As each 400 m lap time was recorded, we were able to accurately assess running speed throughout the race and observed a significant association between GI symptom severity and the reduction in running speed during the final third of the race. From the measurements of real-time GI symptoms and reductions in speed, our data, therefore, suggest that probiotic supplementation could help marathon runners better maintain their running speed, possibly due to the attenuation of GI symptoms. Previously, a ‘gut-training’ protocol that was able to reduce GI symptoms during exercise resulted in an increase in endurance performance, supporting a causal relationship between GI symptom severity and decrements in performance (Miall et al. 2018). However, it is also known that reductions in running speed during the final stages of a marathon can elicit negative emotions (Buman et al. 2008), and therefore, we cannot exclude the possibility that GI symptom scores may have been higher or exaggerated in those runners whom were unable to maintain their race pace. Regardless of the exact reason for the maintenance of race pace, data presented here have shown the potential of probiotic supplementation to improve endurance performance, where GI symptoms are likely to be a deleterious factor.

We (Pugh et al. 2018) and others (Pfeiffer et al. 2012; Ter Steege et al. 2008) have previously reported that 4–27% of runners report ‘moderate’ or ‘serious’ GI symptoms during a marathon race (Pfeiffer et al. 2012; Ter Steege et al. 2008; Pugh et al. 2018). In the current study, 50% of participants reported experiencing one or more moderate symptoms during the race. Interestingly, when specific GI symptoms were assessed immediately post-race, there was no association with these and global in-race symptoms. This discrepancy may present a discourse between measures recorded in real time and measures requiring recall. The higher incidence reported here may also be due to the higher CHO consumption (66 g h^−1^) than ab libitum intakes reported previously (35 ± 26 g h^−1^) (Pfeiffer et al. 2012). This may also explain why studies conducted to date have not shown an association with CHO intake during marathon running and GI symptoms (Hansen et al. 2014; Pfeiffer et al. 2012; Pugh et al. 2018).

As well as an association with GI symptoms and reductions in running speed, there were significant correlations between GI symptoms and post-race sCD14 concentrations, although there were no differences between supplement groups. sCD14 has previously been described as a marker for increased exposure to lipopolysaccharides (Wright et al. 1990), the translocation of which triggers an immune response thought to be an aetiological factor for exercise-induced GI symptoms (Costa et al. 2017a). During a 161 km ultramarathon, plasma sCD14 concentrations were significantly higher post-race in those participants reporting symptoms of nausea (Stuempfle et al. 2016). It should be noted that while sCD14 is stimulated by lipopolysaccharide, it can also be stimulated by a number of toll-like receptor ligands and inflammatory cytokines and so may better be considered a marker of monocyte activation (Shive et al. 2015). However, the lack of any significant correlations between plasma cytokine and sCD14 concentrations in the present study is unsurprising given that strenuous exercise is a strong stimulus for cytokine production, independent of GI damage and GI translocation (Pedersen and Hoffman-Goetz 2000). While we acknowledge that we did not observe an effect of probiotic supplementation on post-race sCD14 concentrations, our data do suggest that sCD14 may be a novel marker for GI-induced immune activation following endurance exercise and provides scope for future research.

There was no association between GI symptoms and LR or I-FABP (used as a marker of GI permeability and damage, respectively), although both were increased post-race. Previously, GI permeability has been shown to be increased following a half and full marathon, but without correlation to any GI symptoms (Smetanka et al. 1999; Oktedalen et al. 1992). Indeed, an inverse correlation between exercise-induced GI permeability and symptoms has also been reported (Costa et al. 2017b), where those with the highest GI permeability reported the lowest symptoms, a finding that has also been found with I-FABP (Costa et al. 2017b). While these markers of GI permeability and damage may have clinical relevance, the lack of relationship with GI symptoms seen here and in the previous studies reinforces both the complexities and difficulties in assessing the deleterious effects of exercise on the GI tract, its ability to retain functionality during endurance exercise, and the mechanisms of individual GI symptoms. Future studies, particularly field-based studies, must, therefore, consider an array of circulatory and subjective measures, although more invasive measures could be needed to fully understand the aetiology of symptoms.

Despite the novel data presented here that adds to the current literature, it is not without some limitations, most of which are due to the using a real-life, competitive marathon race within the study design. Indeed, while the study holds methodological rigor in standardising many of the variables that can affect the incidence of exercise-induced symptoms such as pre-race nutrition, we also acknowledge that the use of an absolute CHO and water intake may not have been the optimum in-race nutrition strategy for each individual. It is known that many components of digestion can vary between individuals such as gastric emptying (Leiper et al. 2001; Rehrer et al. 1992). However, individualised strategies for each participant would have required prior testing, some of which may have been invasive, and so was not possible in the current study. In addition, while participants were instructed to consume all of the contents of each gel, this was not assessed systematically, and therefore, the possibility remains that some participants consumed less and, therefore, may not have reached the prescribed CHO intake. Sweat rates can also vary between athletes (Baker et al. 2016), and therefore, runners may have experienced differing levels of dehydration, which is also known to affect GI symptoms (Lambert et al. 2008; Rehrer et al. 1989). We did not measure core temperature during the race, which has been associated with both GI damage and symptoms (Pires et al. 2016). The cohort here had a large range of finish time, with subsequent ranges in training history and volume. Future studies should also look to recruit participants of more similar characteristics, as these most likely influence the risk of GI symptomology.

In conclusion, we provide novel data demonstrating that probiotic supplementation attenuates GI symptoms during a marathon race, an effect that is associated with the maintenance of running speed during the latter stages of the race. Probiotic supplementation also reduced the frequency of moderate or worse GI symptoms reported, with differences observed after 14 days of supplementation. While we observed differences in these subjective measures, probiotic supplementation had no effect on sCD14, IL-6, IL-8, IL-10, cortisol, or I-FABP concentrations nor or GI permeability (LR). Despite this, we have shown that probiotics offer a promising strategy to reduce the incidence and severity of GI symptoms in endurance runners. Therefore, athletes participating in endurance events, where GI symptoms are common and likely to affect performance could consider probiotic supplementation in the weeks prior to competition.
